# Chemical Cues Induced from Fly-Oviposition Mediate the Host-Seeking Behaviour of *Fopius arisanus* (Hymenoptera: Braconidae), an Effective Egg Parasitoid of *Bactrocera dorsalis* (Diptera: Tephritidae), within a Tritrophic Context

**DOI:** 10.3390/insects11040231

**Published:** 2020-04-07

**Authors:** Pumo Cai, Yunzhe Song, Da Huo, Jia Lin, Huameng Zhang, Zihao Zhang, Chunmei Xiao, Fengming Huang, Qinge Ji

**Affiliations:** 1 Department of Horticulture, College of Tea and Food Science, Wuyi University, Wuyishan 354300, China; caipumo@163.com (P.C.); syz6390@126.com (Y.S.); hawda1090@163.com (D.H.); z15159754369@163.com (H.Z.); Zhangzihao1095@163.com (Z.Z.); XGM2352033@163.com (C.X.); Ulikexx123@163.com (F.H.); 2 Institute of Beneficial Insects, Plant Protection College, Fujian Agriculture and Forestry University, Fuzhou 350002, China; Lin14787861578@163.com; 3 State Key Laboratory of Ecological Pest Control for Fujian and Taiwan Crops, Fuzhou 350002, China; 4 Key Lab of Biopesticide and Chemical Biology, Ministry of Education, Fuzhou 350002, China

**Keywords:** biological control, fruit types, oriental fruit fly, olfactory response, mechanical damage

## Abstract

*Fopius arisanus* is a solitary endoparasitoid that parasitizes a variety of tephritid species. Native to the Indo-Australian region, it is currently exploited worldwide as a biological control agent due to its exceptional efficiency in reducing pest populations. The efficiency of any biological control program is affected by the host location ability of the parasitoids. The present study used a Y-tube olfactometer to test the behavioural responses of female *F. arisanus* to four fruit species which had undergone different types of damages: undamaged, damaged through *Bactrocera dorsalis* ovipositioning (i.e., infested), or different levels of mechanical damage. Our results suggest that *F. arisanus* females were significantly attracted to mangoes and pears (vs. purified air), regardless of their condition; however, whilst infested mangoes did not attract more female parasitoids compared to healthy or mechanically damaged fruits, infested pears attracted significantly more. For citrus fruits and peaches, oviposition damage caused them to be more attractive to parasitoid females. In terms of the longevity of the effects, infested mango fruits remained attractive for up to 5 days after infestation, whereas for infested peaches, pears, and citrus fruits, the attractiveness tended to decrease as time passed. Regarding mechanical damage, mango fruits that had undergone any intensity of damage were equally attractive to parasitoid females; however, peach and citrus fruits with high levels of mechanical damage were more attractive, and pears were found to be most attractive with slight mechanical damage. Additional to the above, we also tested the effect of insecticides on behavioural responses using mangoes. We found that the treatment of infested fruits with lambda-cyhalothrin and cypermethrin remained attractive to *F. arisanus* females, albeit to different extents, which is in contrast to spinosad, cyantraniliprole, and acetamiprid. Finally, we suggest that the host-searching behaviour of *F. arisanus* females is mainly mediated by oviposition-induced volatiles, either emitted from the fruit or left by the fruit fly.

## 1. Introduction:

*Bactrocera dorsalis* Hendle (Diptera: Tephritidae) is one of the most economically important fruit fly pests of Asian origin and infests a wide range of fruit distributed among 46 different plant families [1,2]. In Mainland China, it was first recorded in Hainan in 1934 and has since gradually expanded its distribution range from southern to northern China as a result of global warming and its prominent capacity for rapid reproduction and high spread potential [3]. Today, this polyphagous pest has been documented in almost every region in China [4], where it threatens fruit yield and quality; in southern China, the economic losses amount to approximately three billions USD per year [5]. 

Multiple control tactics incorporating cultural practices, male annihilation, attractant sprays, and biological control, have been explored regarding the integrated management of *B. dorsalis* and other fruit fly species [2,6,7]. Nevertheless, the application of chemical pesticides still plays an essential role in suppressing this pest in the field, which inevitably has detrimental effects on humans, animals, and the environment [7]. The use of parasitoids is an environmentally friendly approach, which is often part of integrated pest management (IPM) programs for the control of frugivorous tephritid fruit fly species [2,8]; its successful application against *B. dorsalis* has been fully demonstrated in the Hawaii islands and French Polynesia [2,8,9,10]. Furthermore, during the outbreak of *B. dorsalis* in Fujian province of China in 2005, the coevolved parasitoid *Fopius arisanus* (Sonan) was used to control the infestation, and was released in combination with several native parasitoids species such as *Psyttalia incisi* (Silvestri), *Diachasmimorpha longicaudata* (Ashmead), and *Fopius vandenboschi* (Fullaway) (Hymenoptera: Braconidae) [11,12].

Indigenous to the Indo-Pacific area, *F. arisanus* is an egg-pupal koïnobiont endoparasitoid considered one of the most effective biological control agents of tephritid pests; it can parasitize the eggs and first instars of approximately 40 Tephritidae pest species [13,14]. *Fopius arisanus* complete their larval and pupal development in the larvae and pupae, respectively, of the pest host [9], and parasitize tephritid pests found on 85 plant species distributed across 35 families [15]. Promising results with *F. arisanus* on *B. dorsalis* have been recorded in southern China: in the field, parasitism rates of 33.3% (in carambola) and 45.8% (in guava) have been reported; in the laboratory, the parasitism rate ranged from 47.4% to 65.7% [16]. The biology of *F. arisanus* has been well studied [17]; however, a few investigations into its chemical ecology have been conducted.

The efficiency of parasitoids as biological control agents heavily relies on their capacity to find suitable hosts [18]. This host-searching behaviour is a sequential process that can be divided into three steps: host habitat location, host location, and host acceptance [19]. During host selection, parasitoids utilize diverse cues involving vibratory, visual, and/or chemical cues, in an interactive pattern. It is widely accepted that chemical cues often play important roles in multiple steps within the host selection process, and generally originate from the phytophagous host and/or the host habitat, and have an effect on both the long- and short-range host searching of parasitoids [20]. Plant-derived cues are more detectable for parasitoids but are a less reliable indication of the occurrence of a suitable host, while host-derived cues are more reliable but less detectable, subjecting foraging parasitoids to a reliability-detectability predicament [21]. Parasitoids have developed various tactics to conquer this difficult situation, one of which is their use of herbivore-induced plant volatiles (HIPVs) emitted from plants as a reaction to herbivore feeding or oviposition [21,22]. These HIPV blends effectively evoke an indirect defence of the host plant by recruiting natural enemies of the herbivore [23]. 

The foraging behaviour of *F. arisanus* on coffee fruits infested by medfly (*Ceratitis capitata* Wiedemann (Diptera: Tephritidae)) eggs has been described by Wang and Messing [24]. Furthermore, numerous studies have examined the relationships between the odour emitted from fly-infested host plants (including fruits and vegetables) and the oviposition behaviour or parasitism rate of fruit fly parasitoids [13,25,26,27,28]. These studies found that the resulting parasitism performance is affected by the fruit or the fruit fly species. Moreover, the attraction of *F. arisanus* to fresh guava and orange fruit odours has been investigated through a wind tunnel [29]; attraction was amplified if the fruits suffered from an infestation of tephritid pests such as the members of the genus *Anastrepha* [14,30]. However, almost all of the above-mentioned studies exposed entire fruits to *F. arisanus*, making it difficult to discriminate whether the effect on host location behaviour was a result of the shape, size, and colour of the fruit, an odour, or a combination of these cues. Furthermore, visual information is often important in host location for parasitoids [20]; *Fopius arisanus* females are significantly more attracted to and landed on objects that were dark yellow rather than objects of other colours [31]. 

However, reports on the infochemicals mediating host locating for *F. arisanus* are scant. The present research was conducted to assess the behavioural responses of female *F. arisanus* to four different fruit species with different treatments in a laboratory olfactory instrument. This study offers more detailed information on the host-searching behaviour of this parasitoid within a tritrophic context and, we hope, will help maximize the control effectiveness of this parasitoid in IPM programs. 

## 2. Material and Methods

### 2.1. Parasitoids (F. arisanus)

An initial strain of *F. arisanus* was established in the Institute of Beneficial Insects, Fujian Agriculture and Forestry University (BII, FAFU) obtained from rotten guava fruits collected in Zhangzhou, Fujian, P.R. China. The climate chamber was held at 25 ± 1 °C and 75% ± 5% relative humidity (RH) with a L:D photoperiod of 14:10 h. Emerged adults were subsequently supplemented with *B. dorsalis* eggs for 10–30 generations. The cohort was kept in a 30 cm × 30 cm × 30 cm Hawaii-type cage [32] following rearing protocols by Manoukis et al. [33]; honey and water were supplied ad libitum. During the earlier-stage rearing of *F. arisanus*, an artificial larval diet without any fruit was provided because the host-searching behaviour of *F. arisanus* is plastic and olfactory preferences for the volatiles that guide them to host patches may change with associative learning [34,35].

From emergence to evaluation, the females used in the following tests were maintained with males in an odourless bioassay room and allowed to acclimate to the conditions; all females were assumed to be mated. 

### 2.2. Fly Colony

Tephritidae species (*B. dorsalis*) were also initially collected from infested guava fruits and reared at BII, FAFU, based on the methods described by Spencer and Fujita [36] under the environmental conditions mentioned above. Eggs were collected using a homemade oviposition bottle containing fruit juice and then transferred to an artificial larval diet composed of wheat bran, torula yeast, sugar, nipagin, sodium benzoate, and water in a fixed ratio; sterilized sand was used as the pupate medium. After pupation, the pupae were collected and shifted to cages where emerged female and male adults were reared together. Adult flies were supplied with water and a food mixture of brown sugar and enzymatic-hydrolysed yeast in a 3:1 ratio ad libitum.

### 2.3. Fruit Materials

Mango, *Mangifera indica* L. (Sapindales: Anacardiaceae); pear, *Pyrus sp.* L.; peach, *Amygdalus persica* L. (Rosales: Rosaceae); and citrus fruits, *Citrus reticulata* Blanco (Rosales: Rosaceae) were purchased from the local organic market. Prior to the experiments, all the tested fruits were washed with water and dried naturally; then, they were bagged under laboratory conditions for several days to ensure the absence of fruit flies. Ripe fruits of a similar size were used.

### 2.4. Behavioural Assays

The olfactory responses of the parasitoids towards volatiles from fruits with different treatments were tested in a modified two-choice olfactometer (all tubes were of 5 cm internal diameter, 15 cm long glass stem, and 15 cm long test glass arms, and had 60° angle in between) that has been previously described [37]. In this apparatus, parasitoids responded to fruit species only via odouriferous cues without the interference of visual or contact bias [38]. The olfactometer system included a small air pump (Thomas 2505N, Shanghai Intelligent Technology Co., Ltd., Shanghai, China), which produced an air stream that first flowed through an activated charcoal filter to clean the air and then through water to humidify the air. Finally, the treated air was divided and pushed through two odour bottles (600-mL glass bottle) which could each hold one tested odour source. The airflow was 200 mL/min per arm. After every replication of each trial, the test arms including the two different odour sources were positioned alternately to remove spatial effects. Furthermore, the test arms and source bottles were replaced with clean ones. Prior to the assay, all glassware were cleaned thoroughly using 75% ethanol and abluent, and thereafter placed in an air-blowing drier at 60 °C for 2 h.

All behavioural bioassays were implemented in a room with uniform lighting to prevent phototaxis and each parasitoid female was tested only once. After releasing the parasitoids into the main stem of the olfactometer, the opening of main stem was blocked by a cotton ball to prevent the parasitoids from escaping. Based on former observations, most parasitoids needed about 30 min to adapt to the circumstances of olfactometer and thereafter chose one of two test arms. A parasitoid that walked beyond one-third the length of either test arm and stayed for over 10 s was considered to select that arm. Only their first choice was recorded. Once all of the parasitoids had made their choices, the trial was ceased. All choice bioassays were applied to this standard criterion for behaviour judgement unless otherwise stated and carried out between 08:00 a.m. and 4:00 p.m.

### 2.5. Experimental Design

#### 2.5.1. Experiment 1: Behavioural Responses to Diverse Fruit Species with Different Treatments

Three different treatments for each fruit type were used: (1) noninfested (undamaged) fruits, (2) fly-infested fruits, and (3) mechanically damaged fruits. Five individual trials for each fruit type were conducted in the Y-tube olfactometer: (1) noninfested fruit vs. purified air, (2) mechanically damaged fruit vs. purified air, (3) fly-infested fruit vs. purified air, (4) noninfested fruit vs. infested fruit, and (5) fly-infested fruit vs. mechanically damaged fruits. To avoid contamination, noninfested fruits were stored in plastic bags with tiny pores to allow air exchange until the start of the choice assay. As for infested fruits, each fruit type was deposited into individual cages (30 cm × 30 cm × 30 cm) containing 10 *B. dorsalis* females of the same age and were allowed to be naturally infested by the fruit fly for 2 h. After infestation, the fruits were removed and observed using a stereomicroscope (TS-50, Beijing PDV Instrument CO. LTD, Beijing, P.R. China) to confirm the occurrence of at least 30 eggs in every fruit. In the third treatment, each fruit type was mechanically damaged using a sterilized entomological needle (size #1; Shandong Hongxiang Plant Protection Tech. CO. LTD, Jinan, Shandong, P.R. China) to resemble damage caused by oviposition of the fly; as the ovipositor length of *B. dorsalis* is approximately 0.4 cm [39], the fruits were punctured up to 25 times to a depth of 0.4 cm. After the treatments, the fruit samples were immediately and randomly placed into odour bottles. Thereafter, one group of 10 parasitoids aged 7–12 days was gently transferred into the opening of the olfactometer and observed until the parasitoids made a choice (generally within 30 min). The numbers of responding parasitoids for each arm were recorded and the response percentage (%) for each arm was calculated as the number of responded parasitoids for one arm divided by the total number of responding parasitoids and multiplied by 100. All tested parasitoids were extracted from the arms by an aspirator irrespective of their choice and were not used for any further tests. This bioassay was replicated 9 times for each trial of each fruit type, thus a total of 1800 parasitoids were used.

#### 2.5.2. Experiment 2: Behavioural Responses to Fruits Infested for Varying Duration

For each trial, each fruit species was naturally infested by *B. dorsalis* as described above. After infestation, all exposed fruits were returned to their bag and preserved for the corresponding duration until the start of the experiment. The ages of infested fruits were: 0 (noninfested host), 1, 2, 3, 4, and 5 days after egg laying. The maximum duration of the treatment was set as 5 days, since after this time, second-instar larvae were likely to be present in the fruit which are not suitable hosts for *F. arisanus*. Two odour bottles for each trial of each fruit type were arranged as follows: (1) noninfested fruit vs. purified air (blank), (2) fruit infested for 1 day vs. purified air (blank), (3) fruit infested for 2 days vs. purified air, (4) fruit infested for 3 days vs. purified air, (5) fruit infested for 4 days vs. purified air, and (6) fruit infested for 5 days vs. purified air. Nine replications were performed for each trial of each fruit type and 1 group of 10 parasitoids was used for each replication, therefore a total of 2160 parasitoids were assayed. The number of responding parasitoids for each arm was recorded, and the relative response percentage of *F. arisanus* females to each fruit species infested for different days was calculated using the following formula: (number of parasitoids responding to fruits − number of parasitoids responding to blank)/(total number of responding parasitoids) × 100. 

#### 2.5.3. Experiment 3: Behavioural Responses to Different Fruits Species with Different Levels of Mechanical Damage

For each fruit species, fruits with mechanical damage of different levels, namely, 0, 25, 50, 100, 200, and 400 punctures, were prepared using a sterilized entomological pin as previously described. Within a dual-choice experiment setup, two odour bottles for each trial of each fruit type were handled as follows: (1) intact fruit vs. purified air (blank), (2) fruit with 25 punctures vs. purified air (blank), (3) fruit with 50 punctures vs. purified air, (4) fruit with 100 punctures vs. purified air, (5) fruit with 200 punctures vs. purified air, and (6) fruit with 400 punctures vs. purified air. Likewise, this experiment was replicated 9 times for each trial of each fruit type and 1 group of 10 parasitoids was used for each replication, therefore a total of 2160 parasitoids were assayed. The numbers of responding parasitoids for each arm were recorded, and the relative response percentage of *F. arisanus* was calculated as per Experiment 2. 

#### 2.5.4. Experiment 4: Behavioural Responses to Mango Fruits Sprayed with Different Insecticides

Five insecticides, namely, lambda-cyhalothrin (v/v: 2.5%, formulation: CS), spinosad (v/v: 2.5%, formulation: SC), cypermethrin (v/v: 10%, formulation: EW), acetamiprid (v/v: 3%, formulation: EC), and cyantraniliprole (v/v: 10%, formulations: SE) were purchased from Jiangsu Changzhou Biochemical Factory, Jiangsu, China and applied strictly under the guidance of the National Minimum Residue Standard of P.R. China. According to the instructions of each pesticide, the five pesticide solutions were individually diluted 2000, 1000, 1500, 1500, and 2000 times, respectively, using water. Infested mango fruits were prepared as mentioned above, and thereafter subjected to surface spraying with 50 uL of each insecticide, respectively, using a microinjector; fly-infested fruits without any insecticide served as the control. The test operation was the same as the above dual-choice experiments. Within one trial of each insecticide type, one odour bottle containing infested mango without any pesticide served as the control; the other bottle containing infested and insecticide-treated mango was the treatment group. Similarly, this experiment was replicated 15 times for each insecticide type and 1 group of 10 parasitoids was used for each replication, therefore a total of 750 parasitoids were assayed. The response percentage of *F. arisanus* was calculated as the number of parasitoids responding to the pesticide-treated group minus the number of parasitoids responding to the free-pesticide group and divided by the total number of responding parasitoids and finally multiplied by 100.

#### 2.5.5. Experiment 5: Which Factor Plays a More Important Role in *F. arisanus* Host Location

Finally, we used the Y-tube olfactometer to examine whether parasitoid orientation to fruits was influenced by eggs hidden within the fruits (using only peach and citrus fruits) or due to physical damage caused by flies’ oviposition puncture. Hence, four different treatments were established: (1) fruit exposed to fertile *B. dorsalis* females; (2) fruit exposed to sterile *B. dorsalis* females; (3) fruit with physical damage but without egg implantation; and (4) fruit with physical damage and eggs manually inserted inside the fruit. Purified air was used as the control group in this dual-choice experiment. Treatments 1 and 3 were prepared in line with the procedure described in Experiment 1. According to a previous report by Jayanthi et al. [40], sterile *B. dorsalis* females exhibit the same oviposition behaviour as fertile ones but do not deposit eggs. In treatment 4, *B. dorsalis* eggs were implanted in pierced fruits using a small hairbrush. This was replicated 9 times for each trial and 1 group of 10 parasitoids was used for each replication, therefore a total of 360 parasitoids were assayed. The relative response percentage of *F. arisanus* was calculated as per Experiment 2.

### 2.6. Data Analysis

Statistical analyses were performed using SPSS 17.0 (SPSS, Inc., Chicago, IL, USA) and GraphPad Prism 7.0 (GraphPad Software, San Diego, CA, USA). The data from Experiment 1 for each treatment were analysed using Chi-square tests and Experiments 2–5 were subjected to one-way analysis of variance (ANVOA) followed by Tukey’s honestly significant difference test (HSD) test (*p* < 0.05) for multiple mean comparisons. All percentage data were square root transformed prior to analysis to enhance normality and homoscedasticity [41]. However, untransformed data are exhibited in the figures. 

## 3. Results

### 3.1. Experiment 1: Behavioural Responses to Diverse Fruit Species with Different Treatments

**Figure 1:**
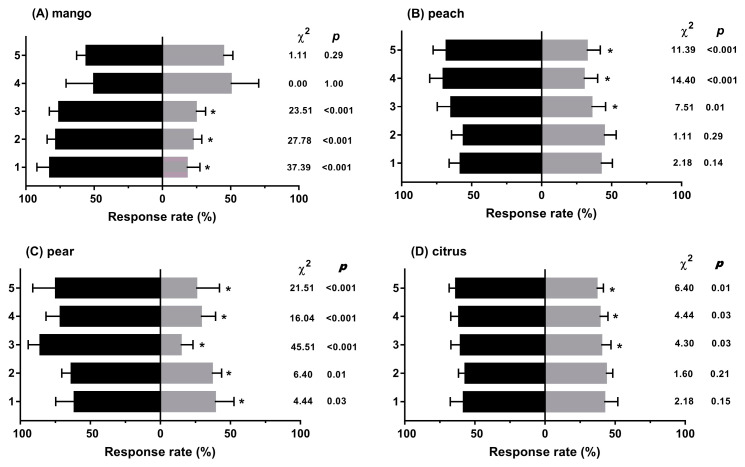
Behavioural responses of *F. arisanus* females to diverse fruit species with different treatments under a dual choice test. (**A**) mango, (**B**) peach, (**C**) pear, (**D**) citrus. The data are expressed as mean ± SD. Numbers 1 to 5 in the Y-axes of all panels refer to: (**1**) noninfested fruit vs. purified air; (**2**) mechanically damaged fruit vs. purified air; (**3**) infested fruit vs. purified air; (**4**) infested fruit vs. noninfested fruit; (**5**) infested fruit vs. mechanically damaged fruit (black bar vs. grey bar, respectively). Asterisks denote statistical differences using a Chi-square test (*p* < 0.05). N = 9.

Mangoes, whether intact or not, were found to significantly attract *F. arisanus* females: they significantly responded to infested, intact, and mechanically damaged mango fruits compared to purified air, and their response to infested mangoes was not significantly different to that for intact or mechanically damaged fruit (Figure 1A). As for peach and citrus fruits, *F. arisanus* females were not significantly attracted to noninfested or mechanically damaged fruit; however, fruits infested by *B. dorsalis* were obviously attractive for *F. arisanus* in comparison to healthy fruits, mechanically damaged fruits, or purified air (Figure 1B,D). Noninfested pears were significantly attractive to *F. arisanus* females, which was evidently enhanced by both mechanical damage and infestation, the latter of which was the most attractive (Figure 1C). 

### 3.2. Experiment 2: Behavioural Responses to Fruits Infested for Varying Duration

**Figure 2:**
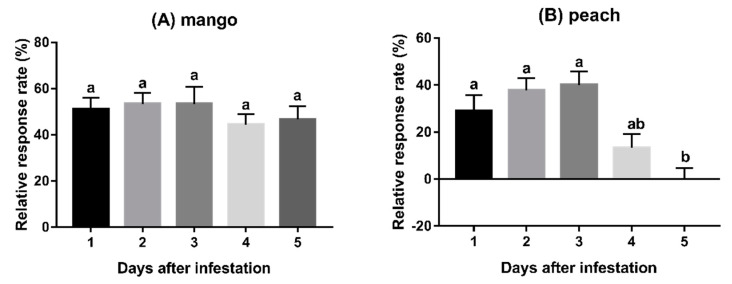

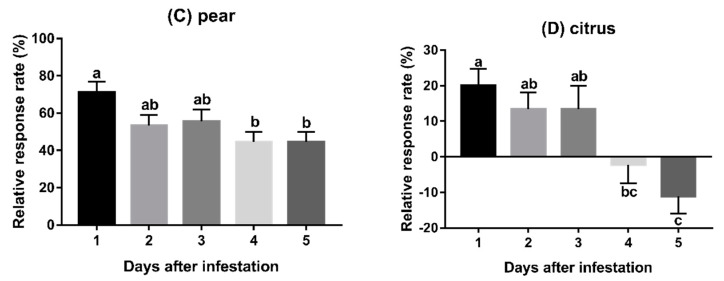
Behavioural responses of *F. arisanus* females to fruits infested for different numbers of days. (**A**) mango, (**B**) peach, (**C**) pear, (**D**) citrus. The data are expressed as mean ± SD. Different lowercase letters above bars denote significant differences by Tukey’s multiple range test (*p* < 0.05). N = 9.

The behavioural responses of *F. arisanus* females to infested mangoes did not significantly vary with the number of days after fly egg deposition; all durations were equally as attractive to parasitoids (Figure 2A, *F_4,40_* = 0.53, *p* = 0.72). Peach and citrus fruits that had been infested by *B. dorsalis* eggs for 5 days were significantly less attractive to *F. arisanus* females compared to 1–3 days (Figure 2B,D; peach: *F_4,40_* = 5.31, *p* = 0.002; citrus fruits: *F_4,40_* = 5.93, *p* = 0.001). Infested pears were attractive to *F. arisanus* females throughout the 5 days duration; nevertheless, significant differences existed between first day after infestation and the fourth or fifth days (Figure 2C, pear: *F_4,40_* = 3.48, *p* = 0.016).

### 3.3. Experiment 3: Behavioural Responses to Different Fruit Species with Different Levels of Damage

**Figure 3:**
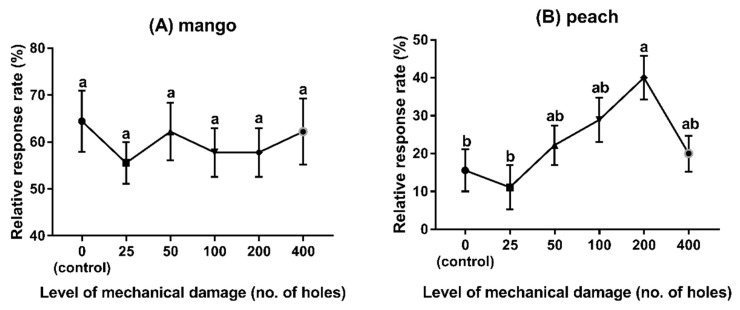

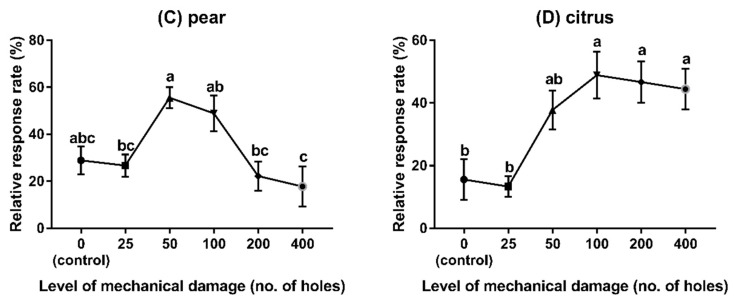
Behavioural responses of *F. arisanus* females to different fruits species with different levels of mechanical damage. (**A**) mango, (**B**) peach, (**C**) pear, (**D**) citrus. The data are expressed as mean ± SD. Different lowercase letters denote significant differences by Tukey’s multiple range test (*p* < 0.05). N = 9.

As shown in Figure 3A, the behavioural responses of *F. arisanus* females to mango fruits were not influenced by the level of mechanical damage *(F_5,48_* = 0.35, *p* = 0.88). However, the attraction to peaches with 200 punctures was significantly higher than to those with 0 or 25 punctures (Figure 3B, peach: *F_5,48_* = 3.48, *p* = 0.009) and citrus fruits with 100, 200, and 400 punctures attracted significantly more parasitoids compared to those with 0 or 25 punctures (Figure 3D, citrus fruits: *F_5,48_* = 6.51, *p* < 0.01). For pears, the highest attraction was obtained with 50 punctures on the surface, which was found to be significantly more attractive than fruit with 25 or 200–400 punctures (Figure 3C, pear: *F_5,48_* = 5.75, *p* < 0.01).

### 3.4. Experiment 4: Behavioural Responses to Mango Fruits Sprayed with Different Insecticides

**Figure 4:**
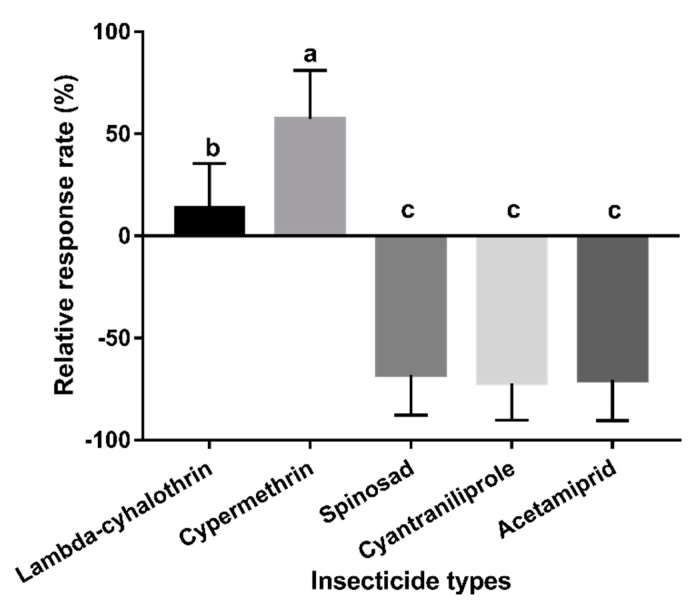
Behavioural responses of *F. arisanus* females to fly-infested mangoes sprayed with different insecticides. The data are expressed as mean ± SD. Different lowercase letters denote significant differences by Tukey’s multiple range test (*p* < 0.05). N = 15.

Using exclusively mango fruits, the relative response rates of parasitoid females to fly-infested fruits sprayed with different insecticides were significantly different (*F_4,20_* = 123.882, *p* < 0.01). The parasitoids showed a preference for fruits sprayed with lambda-cyhalothrin and cypermethrin (vs. control), although to different extents. This is in contrast to fruits sprayed with spinosad, cyantraniliprole, and acetamiprid (Figure 4), whereby the control was preferred. 

### 3.5. Experiment 5: Which Factor Plays a More Important Role in F. arisanus Host Location?

**Figure 5:**
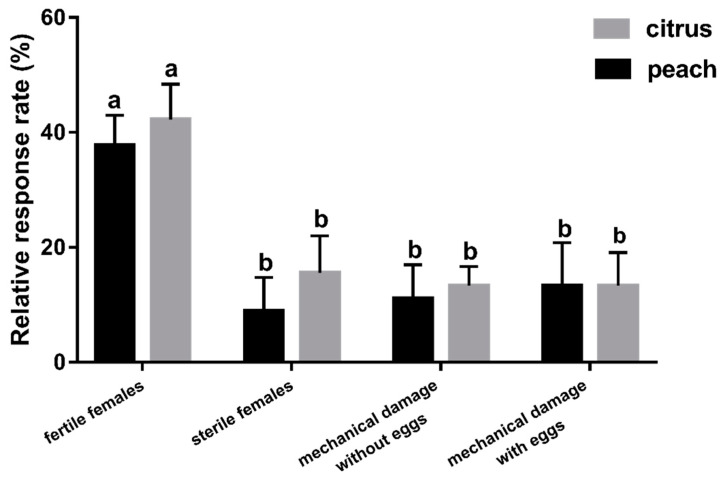
Behavioural responses of *F. arisanus* females to two fruits exposed to fertile and sterile *B. dorsalis* females, mechanically damaged fruits without eggs and mechanically damaged fruits with egg embedding. The data are expressed as mean ± SD. Different lowercase letters denote statistical differences by Tukey’s multiple range test (*p* < 0.05). N = 9.

Female *F. arisanus* were more attracted to both peach and citrus fruits exposed to fertile *B. dorsalis* females than to fruits exposed to the other treatments (i.e., sterile females (no eggs); mechanically damaged fruits, without inserting eggs; mechanically damaged fruits, with inserting eggs inside punctures) (Figure 5; peach: *F_3,32_* = 4.77, *p* = 0.007; citrus fruits: *F_3,32_* = 6.39, *p* = 0.002).

## 4. Discussion

In the present context, the host pest is concealed within the plant. The fact that the host is effectively “invisible” requires the parasitoid seek and accept not only the host pest but also the host’s habitat [42]. As such, parasitoids have evolved diverse strategies to search for well-hidden hosts and have the ability to discriminate between plants (as shown through their olfactory preferences) that are hosts of their hosts [42,43]. Physical characteristics and infochemicals from host plants are well-known as the main cues that influence the orientation behaviour of parasitoids, such as seeking for oviposition sites, mating sites, and nutrition [15]. In our study, a Y-tube olfactometer was used to eliminate the effects of visual and touch cues; only infochemicals emanating from host eggs or host fruits were considered. 

In the case of chemical cues, our study suggests that *F. arisanus* females may use volatiles emitted from healthy fruits during the host location process. This is in line with previous research [14,25,29] which demonstrated that female *F. arisanus* were attracted to odours emitted from healthy host fruits (mango, orange, and guava), hosts crops for ecologically different tephritid species (zucchini, tomato, and Indian almond), and a nonhost fruit (strawberry). Nevertheless, not all healthy host fruits could attract *F. arisanus* females, such as with the peach and citrus fruits tested in this study, even if they were pierced artificially prior to the experiment. Few studies have investigated infochemicals emanating from fresh host plants; consequently, further studies are needed to quantitatively compare the differences in chemical composition between various fruit types which cause distinctive behaviour of parasitoids to clarify the role of major compounds in the orientation of *F. arisanus*.

Our results also suggest that infested peach, pear, and citrus fruits were significantly more attractive to *F. arisanus* females than those not infested by *B. dorsalis* eggs. However, for mango, this was not observed. This is in contrast with the findings of Rousse et al. [14] who found that mangoes infested with the eggs of *Bactrocera zonata* Saunders (Diptera: Tephritidae) were more attractive than uninfested fruits. It is important to note, however, that although both experiments used the same parasitoid and host plant types, they differ in host species. Each fruit fly species will stimulate the emission of different HIPVs, which may generate a different response in the parasitoid [44]. The stronger attraction to a higher trophic level observed in infested fruits compared with uninfested ones could be due to synomones elicited by the infestation or kairomones left by the host [45]. This also demonstrates that *F. arisanus* females are capable of distinguishing semiochemicals emitted from specific fruits which are either infested or noninfested [46].

Infested mango fruits remained attractive to *F. arisanus* females up to and including 5 days after infestation, after which they became rotten and contained only second- or later-instar larvae (which *F. arisanus* cannot parasitize). Surprisingly, we found that *F. arisanus* females were still attracted to decaying mango fruits containing only a limited number of fruit fly eggs. We suspect that this is because the occurrence of rotten fruits could indicate to the parasitoids the vicinity of fresher ones or damaged pests. A different scenario was found for the other fruits (i.e., peach, pear, and citrus fruits) whereby they became less attractive to this parasitoid as time postinfestation passed, and citrus fruits exhibited a repelling effect 5 days postinfestation. This feature could prove useful in the development of parasitoid-based biological control methods in different orchards, particularly in optimizing the timing of parasitoid release. Furthermore, our laboratory findings may help to explain the field situation where it has been found that *F. arisanus* females prefer to search host fruits suspended from the tree rather than the rotten fruits on the ground [2,47,48].

Numerous studies have compared mechanical damage with herbivore damage and have investigated how this affects the response of parasitoids [49]; however, a few reports have documented the effect of oviposition damage. In Experiment 1, our results suggested that *F. arisanus* females did not discriminate between mechanical damage and oviposition damage for mango fruits, but they were able to differentiate between these two types of damage for peach, pear, and citrus fruits. Moreover, it is interesting that *F. arisanus* exhibited distinct olfactory orientation trends as the intensity of mechanical damage increased for each of the fruit species. For mangoes, it was found that there was no significant variation in attraction to parasitoids for the different levels of damage. For peaches and citrus fruits, however, parasitoids were greatly attracted to highly damaged fruits (200 punctures and 100–400 punctures, respectively). For pears, the greatest attraction was found at a low-intensity of mechanical damage (50 punctures). We hypothesise that this variation in behavioural response is due to qualitative and quantitative differences in the volatile blend release, which are worthy of further study.

In the present study, infested fruits sprayed with given dose of lambda-cyhalothrin and cypermethrin exerted different levels of attractiveness to *F. arisanus* females, whereas spinosad, cyantraniliprole, and acetamiprid had the opposite effect. This is in line with a previous study which suggested that spraying cypermethrin on tobacco leaves resulted in a positive effects on the foraging behaviour of *Aphidius gifuensis* Ashmead (Hymenoptera: Braconidae), whereas tobacco leaves treated with 20% imidacloprid, 3% acetamiprid, or 40% omethoate repelled this parasitoid in a laboratory test [50]. Previous research also revealed that exposing parasitoids to the LD_20_ (the dose that induces 20% mortality) of the insecticide chlorpyrifos in the presence of hosts and bananas improved the percentage of female *Leptopilina heterotoma* Thomson (Hymenoptera: Eucoilidae) responding to the banana odour and enhanced the extent and duration of probing activity [51]. The plasticity of host location behaviour in this parasitoid could be used to develop a cohort that have good performance in the presence of insecticide [14,34]. Plant volatiles are mainly generated from the plant’s secondary metabolism; their synthesis and release are influenced by biotic and abiotic factors in nature, and can be affected by insecticides. For example, terpenes, which are a constituent of plant volatiles, are biosynthesized by the isoprene pathway, and the initial reactants of this pathway are leucine, valine, and acetyl coenzyme A [52]. A previous research has found that organochlorine insecticides can increase or decrease the leucine and valine content in plants [53]. This may eventually lead to a change in the plant volatiles, which will subsequently affect the behaviour of natural enemies during host searching, which may explain our result.

In Experiment 5, we corroborated that *F. arisanus* females prefer fruits infested with fertile female *B. dorsalis* (i.e., hiding eggs) over fruits punctured by sterile *B. dorsalis* females, mechanically damaged fruits, or mechanically damaged fruits with artificially inserted eggs. Ji et al. [5] found that nine components of infochemicals from the surface of *B. dorsalis* eggs could induce different levels of electroantennogram (EAG) and female parasitoid behavioural responses. Theoretically, mechanically damaged fruits with egg insertion could result in a similar level of attraction to fruits exposed to fertile *B. dorsalis* females; however, our results show otherwise. We therefore suppose that the host location behaviour of *F. arisanus* females is mediated by infochemicals emanating from fruits specifically in response to oviposition. Previous studies have found that some tephritid pests deposit host marking pheromones (HMP) which are known to act as kairomones for opiine parasitoids [54] near the egg-laying site [55]. This kind of pheromone is only produced by ovipositing tephritid females [14], fully supporting our explanation that female *F. arisanus* utilize oviposition-induced volatiles during host location. The chemical identification of the infochemicals induced from oviposition activity and the underlying mechanisms that stimulate the release of volatiles require further investigation.

## 5. Conclusions

The present research examined how *F. arisanus* females respond to four different fruits under various treatment scenarios and investigated whether the fruit volatiles that attract this parasitoid are induced as a result of the oviposition activity of *B. dorsalis*. Our main findings suggest that the different fruits exhibited distinct strategies for recruiting *F. arisanus* females after they had been damaged either mechanically or by the oviposition of *B. dorsalis*. Moreover, oviposition-induced volatiles from fruits or pheromones deposited by ovipositing fruit flies may play an important role in the host-searching behaviour of *F. arisanus* females. Interestingly, insecticides such as lambda-cyhalothrin and cypermethrin both imposed different levels of attraction to parasitoids. This information could be considered as a foundation and reference for the application of *F. arisanus* in the field and to amplify their effectiveness in biological control programmes to suppress *B. dorsalis* populations.
